# Cinnamomi Cortex (*Cinnamomum verum*) Suppresses Testosterone-induced Benign Prostatic Hyperplasia by Regulating 5α-reductase

**DOI:** 10.1038/srep31906

**Published:** 2016-08-23

**Authors:** Hyun-Myung Choi, Yunu Jung, Jinbong Park, Hye-Lin Kim, Dong-Hyun Youn, JongWook Kang, Mi-Young Jeong, Jong-Hyun Lee, Woong Mo Yang, Seok-Geun Lee, Kwang Seok Ahn, Jae-Young Um

**Affiliations:** 1 College of Korean Medicine, Basic Research Laboratory for Comorbidity Regulation, Kyung Hee University, 26 Kyungheedae-ro, Dongdaemun-Gu, Seoul, 130-701, Republic of Korea; 2 Department of Science in Korean Medicine, Graduate School, Kyung Hee University, 26 Kyungheedae-ro, Dongdaemun-Gu, Seoul, 130-701, Republic of Korea; 3 College of Pharmacy, Dongduk Women’s University, 60 Hwarang-ro 13-gil, Seongbuk-gu, Seoul, 136-714, Republic of Korea

## Abstract

Cinnamomi cortex (dried bark of *Cinnamomum verum*) is an important drug in Traditional Korean Medicine used to improve blood circulation and Yang Qi. Benign prostatic hyperplasia (BPH) is a common chronic disease in aging men. This study was conducted to determine the effect of Cinnamomi cortex water extract (CC) on BPH. BPH was induced by a pre-4-week daily injection of testosterone propionate (TP). Six weeks of further injection with (a) vehicle, (b) TP, (c) TP + CC, (d) TP + finasteride (Fi) was carried on. As a result, the prostate weight and prostatic index of the CC treatment group were reduced. Histological changes including epithelial thickness and lumen area were recovered as normal by CC treatment. The protein expressions of prostate specific antigen, estrogen receptor α (ERα), androgen receptor (AR), 5α-reductase (5AR), and steroid receptor coactivator 1 were suppressed by treatment of CC. Immunohistochemical assays supported the western blot results, as the expressions of AR and ERα were down-regulated by CC treatment as well. Further *in vitro* experiments showed CC was able to inhibit proliferation of RWPE-1 cells by suppressing 5AR and AR. These results all together suggest CC as a potential treatment for BPH.

Benign prostatic hyperplasia (BPH) is a common chronic disease in aging men^1^,^2^. The incidence of BPH increases with age. In males over 60 years of age, more than 50% of them suffer from BPH symptoms^3^.

BPH is mainly caused by proliferations of smooth muscle and epithelial cells locating in the prostate. Growth of the prostates leads to lower urinary tract symptoms (LUTS) which include urinary intermittency, frequency, straining, urgency, weak stream, incomplete emptying, and nocturia^4^.

Although the etiology of BPH is unknown, testosterone and dihydrotestosterone (DHT) are well-known to be related to the development of BPH. Testosterone which is produced in the testis spreads to the prostate, and the testosterone is converted into DHT by the action of 5α-reductase (5AR), an enzyme involved in steroid metabolism^5^,^6^. DHT is five times more important than testosterone to expression of BPH^7^. While serum concentration of testosterone decreases with age, the activities of 5AR and androgen receptor (AR) are increased due to androgen balance. High concentration of DHT enhances prostate specific antigen (PSA) levels by binding with AR. The PSA levels can be increased by increased prostate volume or inflammation. Despite the fact it is not a unique indicator of prostate cancer, PSA is widely used to help the diagnosis of BPH^8^,^9^.

Estrogen exists in the prostate and has effects on the development of BPH. Estrogen receptor α (ERα) and estrogen receptor β (ERβ) are differently expressed and contrarily functioned: ERα mediates cell proliferation, but ERβ mediates apoptosis^10^,^11^. Thus, ERβ has beneficial effects for repressing the growth of the prostate. On the other hand, the function of ERα may possibly grow the size of the prostate^12^. As well as AR and ERs, various co-regulators interact directly with AR resulting in enhancement or reduction of its transcriptional activity. Among several reported co-regulators, one of the most extensively characterized AR co-regulator is the steroid receptor coactivator 1 (SRC1), which can affect AR-mediated cell proliferation^13^.

BPH patients share common features: proliferated prostate cells and increased DHT, AR, and PSA levels. Patients who suffer from LUTS need surgery or medication. The most broadly used medications are α-blockers and 5α-reductase inhibitors (5ARIs). Alpha-blocker is an α_1_-adrenergic receptor antagonist^14^. In the case of BPH, they are useful because of their ability to relax the smooth muscles in the prostate and the bladder neck, as a result helping urine flow^15^,^16^. However, α-blockers have limitations; they are not capable of reducing the size of the prostate, and also show side effects such as orthostatic hypotension, ejaculation changes, headaches, nasal congestion, and weakness^17^,^18^. 5ARIs such as finasteride (Fi) which suppresses 5AR-2 or dutasteride which suppresses both 5AR-1 and 2 are prescribed to inhibit testosterone’s exchange to DHT and prevent the combining of AR, and also reduce PSA, all as a result of inhibition of 5AR^19^,^20^. But 5ARIs also exhibit side effects including decreased libido and ejaculatory or erectile dysfunction^21^. Therefore, herbal medicines are considered for treatment of BPH. Herbal medicines have lower side effects in general, so many BPH patients are exploring use of complementary and alternative medicine.

Cinnamomi cortex is the dried inner bark of *Cinnamomum verum*. In ancient Asia, Cinnamomi cortex was used as a very important spice and drug which has contributed to improving blood circulation and Yang Qi in the kidney^22^. Previous studies reported Cinnamomi cortex^23^ shows antioxidant activity, and keishibukuryogan, a herbal formula including Cinnamomi cortex, has benefit to reducing renal damage in diabetic rats^24^. However, the effects of Cinnamomi cortex on BPH have not been established. Therefore, this study is aimed to investigate the effects of the water extract of Cinnamomi cortex (CC) on prostatic hyperplasia using a testosterone propionate (TP)-induced BPH rat model, and reveal the mechanisms of the action involved in its activity.

## Results

### GC-MS analysis of CC

**Figure 1:**
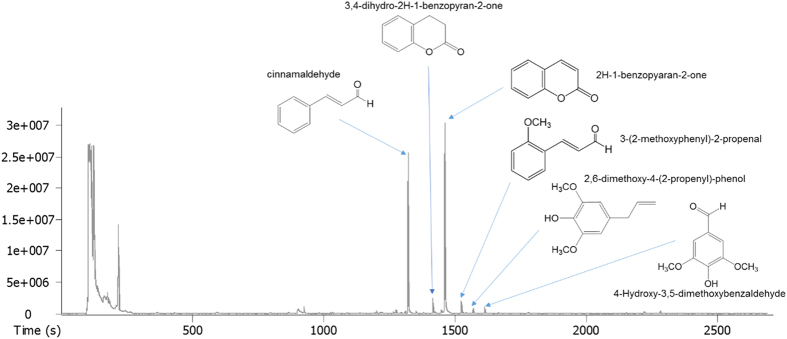
Total ion chromatogram of extract of CC by GC-MS and chemicals identified with a library.

When the extract was analyzed by GC-MS and compared with a library, cinnamaldehyde, 3,4-dihydro-2H-1-benzopyran-2-one, 2H-1-benzopyaran-2-one, 3-(2-methoxyphenyl)-2-propenal, 2,6-dimethoxy-4-(2-propenyl)-phenol and 4-hydroxy-3,5-dimethoxy-benzaldehyde were mainly detected (Fig. 1).

### Effects of CC on prostate weight and prostate weight index in TP-induced BPH rats

**Figure 2:**
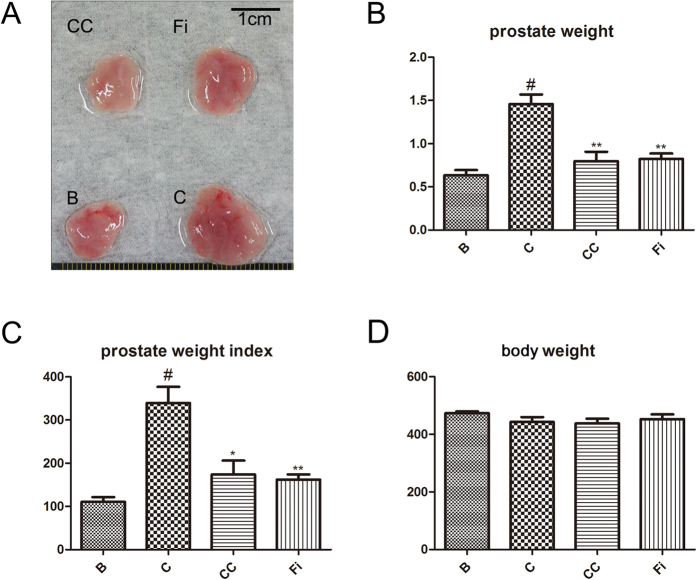
Effect of CC on prostate weight and prostate index in TP-induced BPH rats. (**A**) The dissection of prostates. (**B**) The total prostate weight of the rats. (**C**) Prostate weight index. (**D**) The body weight of the rats. The prostate indexes were calculated dividing prostate weight (mg) by body weight (100 g). ^#^*P* < 0.05 when compared to B; *P < 0.05 when compared to C. B, normal control group; C, TP-induced BPH group; CC, CC treated BPH group; Fi, Fi treated BPH group.

Changes in prostate tissues in control group and experimental group are shown in Fig. 2. The body weight, total prostate weight, and prostate weight index (PW index, mg of prostate per 100 g body weight) are shown in Table 1. When the rats were treated TP, they showed significant increase in prostate weight (1456 ± 196 mg) and PW index (339.4 ± 64.8) compared to the negative control group (prostate weight 473.1 ± 15.6 mg, PW index 111 ± 18.3) (*P* < 0.05). The positive control group treated with Fi also showed significant changes in prostate weight (823 ± 33.2 mg) and PW index (162.3 ± 20.8) both (*P* < 0.05). The PW index was recovered in CC group by 48.8% and in Fi group by 52.2%. There were no significant changes in body weight.

### Effects of CC on epithelial thickness in TP-induced BPH

**Figure 3:**
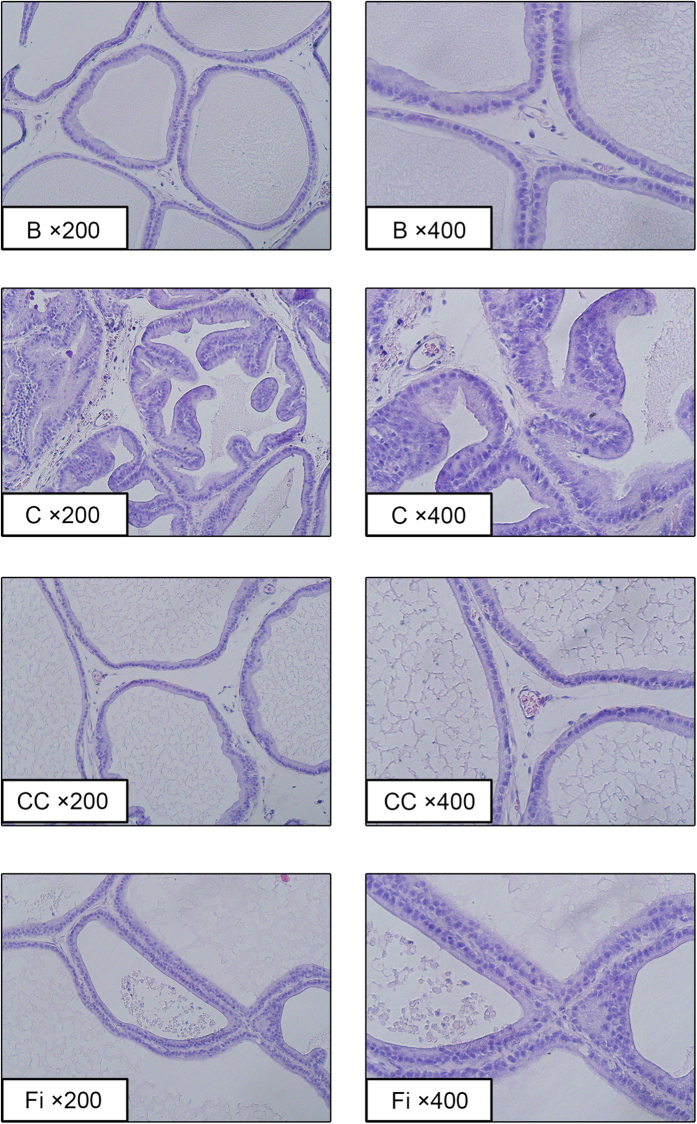
Effect of CC on histological changes of the prostate tissues in TP-induced BPH rats. Representative photomicrograph of H&E stained prostate tissues (left panel magnification × 200, right panel magnification × 400). B, normal control group; C, TP-induced BPH group; CC, CC treated BPH group; Fi, Fi treated BPH group.

Figure 3 shows the histological changes of prostate tissues. Histological changes including epithelial thickness and lumen area were recovered as the normal group by CC treatment. TP-treated BPH group (control) showed epithelial hyperplasia compared with normal control (blank). CC treated group and Fi treated group also showed reductions in epithelial thickness.

### Effect of CC on protein expressions of PSA, 5AR-2, ERα, AR, and SRC1 in prostate tissues in TP-induced BPH rats

**Figure 4:**
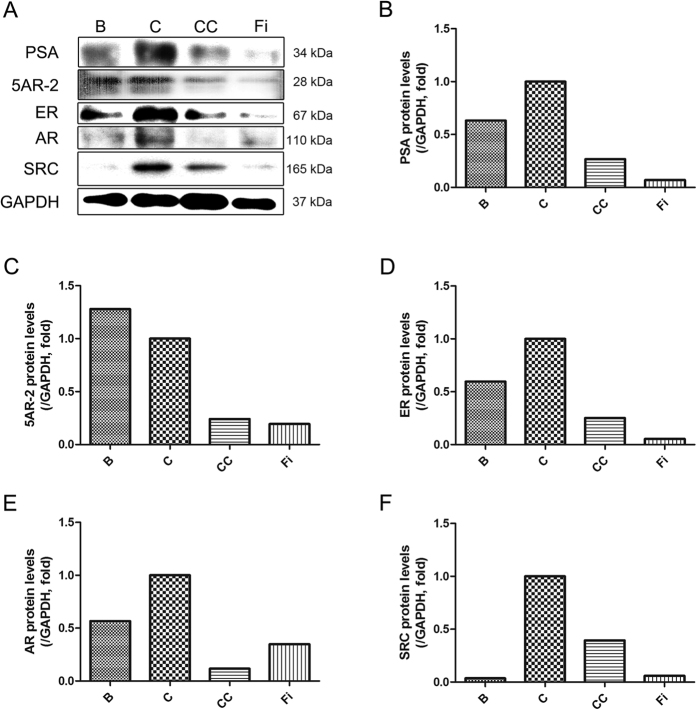
Effect of CC on protein expressions of PSA, 5AR-2, ER-α, AR, and SRC1 in the prostate tissues of TP-induced BPH rats. The relative protein expressions of PSA, 5AR-2, ERα, AR, and SRC1 were analyzed by the western blot analysis. The protein expressions differences were normalized to GAPDH. Values are mean ± S.D. of data from three separate experiments. B, normal control group; C, TP-induced BPH group; CC, CC treated BPH group; Fi, Fi treated BPH group.

In the pathogenesis of BPH, testosterone is converted into DHT by the action of 5AR-2 in the prostate^5^,^6^. This specific hormone, DHT, binds to AR and AR-coactivators such as SRC1, and then induces proliferation of prostate cells^13^,^20^ with elevated PSA levels^8^,^19^. On the other hand, activated ERα also results in prostatic enlargement^10^,^11^,^12^. Figure 4 shows the expression of PSA, 5AR-2, ERα, AR, and SRC1 by western blot analysis. The protein levels of PSA, 5AR-2, ERα, AR, and SRC1 were increased by the TP-treated group (control) compared to the normal control group (blank). However, the expressions of PSA, 5AR-2, ERα, AR, and SRC1 proteins were down-regulated by CC when compared with the TP-induced BPH group. Especially, AR and SRC1 were more inhibited in CC group than Fi group.

### Effect of CC on expression of AR in prostate tissues in TP-induced BPH rats

**Figure 5:**
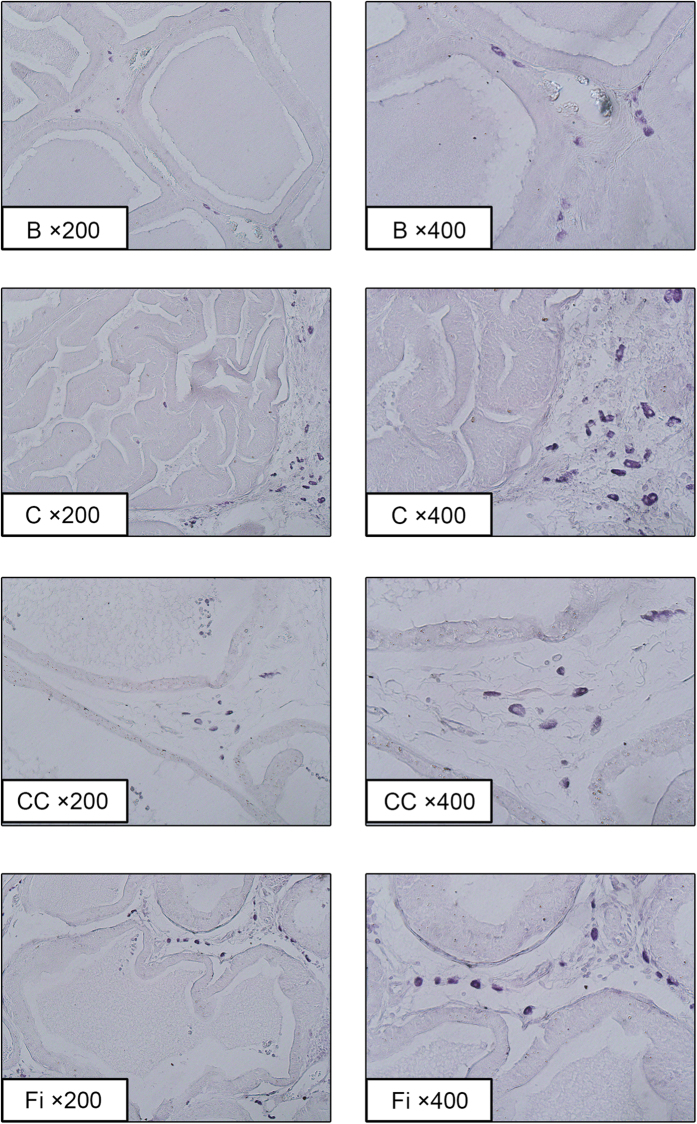
Immunohistochemical analysis of AR in prostate tissues of TP-induced BPH rats. Representative photomicrographs of the prostate tissues (left panel magnification × 200, right panel magnification × 400). The stained dots indicate anti-AR immunostained cells. B, normal control group; C, TP-induced BPH group; CC, CC treated BPH group; Fi, Fi treated BPH group.

AR, which is to play an important role in the development of BPH, was evaluated by microscopic examination of the prostate tissues immunostained by Anti-AR Ab. TP-treated group showed elevated expression compared with the normal control group, but CC suppressed the expression of AR (Fig. 5).

### Effect of CC on expression of ERα in prostate tissues in TP-induced BPH rats

**Figure 6:**
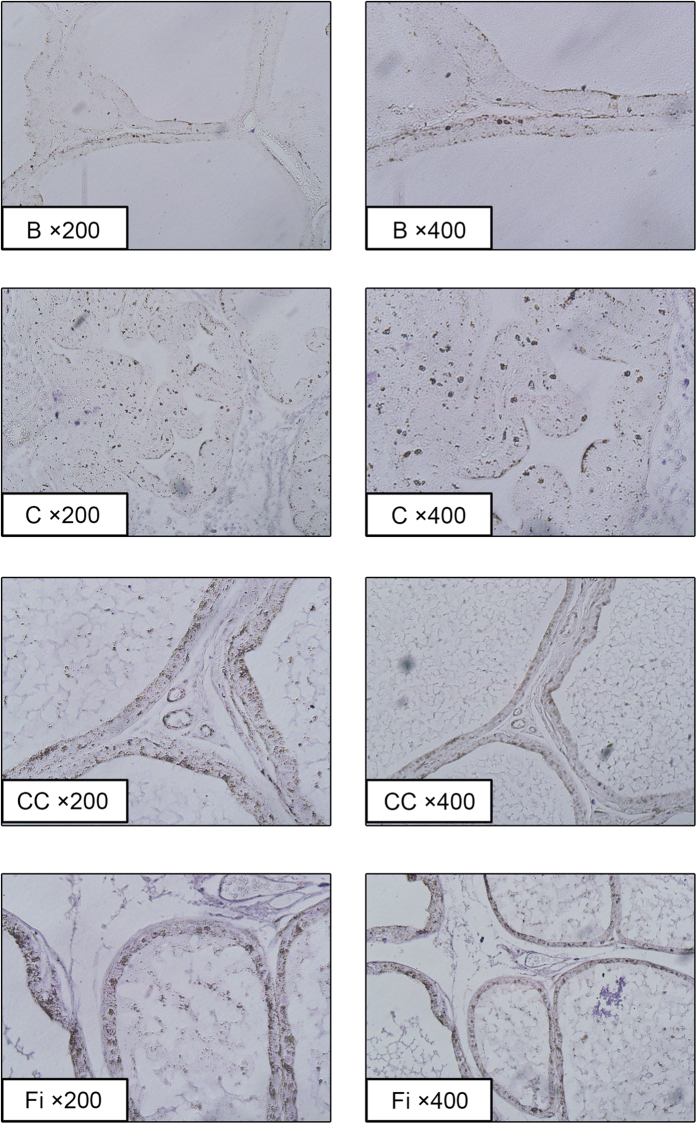
Immunohistochemical analysis of ERα in prostate tissues of TP-induced BPH rats. Representative photomicrographs of the prostate tissues (left panel magnification × 200, right panel magnification × 400). The stained dots indicate ERα immunostained cells. B, normal control group; C, TP-induced BPH group; CC, CC treated BPH group; Fi, Fi treated BPH group.

ERα is known to involve in prostate growth, inflammation, and dysplasia. In BPH patients, levels of ERα are higher than normal males. ERα expression was evaluated by microscopic examination of the prostate tissues immunostained by Anti-ERα Ab (Fig. 6). TP-treated group showed elevated expression compared with normal control group. Similar to the AR expression results, CC treated group and Fi treated group showed down-regulated expressions of ERα.

### Effect of CC on RWPE-1 prostate epithelial cells

The normal prostate epithelial cell-line, RWPE-1, was used in order to performed *in vitro* studies. An MTS assay was conducted in order to measure the effects of CC on proliferation of RWPE-1 cells. As a result, CC inhibited 24 h cell proliferation of RWPE-1 at the concentration of 500 and 1000 μg/ml by 85.12% and 68.97% respectively, compared to non-treated control cells (see Supplementary Fig. S1).

Further western blot assays were carried on to investigate the proliferation inhibiting mechanism. Despite the fact 5 and 50 μg/ml of CC could not inhibit proliferation of RWPE-1 cells in the MTS assay, the protein expressions of PSA, AR and 5AR were suppressed at the all of the concentrations of CC (5, 50, and 500 μg/ml), suggesting even low doses which did not affect cell proliferation were able to decrease expressions of 5AR and AR, and this down-regulated levels of 5AR and AR lead to inhibition of RWPE-1 proliferation when higher dose was used (see Supplementary Fig. S2).

## Discussion

In this study, the authors investigated the effect of CC in TP-induced BPH rat model of Kato *et al*.^25^, which was improved by Maggi and colleauges^26^ and is currently used widely as a novel *vivo* model for BPH studies^27^,^28^. Based on the detailed human study of McNeal^29^, several homologies offer an opportunity to examine animal BPH models with the premise of understanding the mechanisms and etiology of pathological processes involved in BPH. Administration of TP induced enlargement of the size of the prostate and increased the protein levels of PSA, 5AR-2, ERα, AR, and SRC1. However, CC-treated model showed a reduction in size of prostate and the decreases of PSA, 5AR-2, ERα, AR, SRC1 protein levels. In addition, we have showed that the AR and ERα expression were down-regulated by CC treatment when analyzed by immunohistochemical staining of prostate tissues.

The main types of medicines for an enlarged prostate are α-blockers and 5ARIs. Alpha-blockers including doxazosin, terazosin, tamsulosin, and alfluzosin relax the muscles in the prostate and around the neck of the bladder and they make easier to pass urine. Alpha-blockers are often used as the first step of medicines^30^, but they cannot reduce the size of the enlarged prostate^31^. In addition, they have some side effects; first dose syncope, dizziness, tachycardia, hypotension, headache, asthenia, rhinitis, and ejaculatory dysfunction^32^. In contrast, 5ARIs such as finasteride and dutasteride, reduce the size of the prostate gland, taking pressure off the urethra and making it easier to pass urine. They can shrink the prostate gland by up to a quarter (20 to 30 percent) after 6 to 12 months of treatment. Even though there were some reports of successful treatment outcomes after long-term use of 5ARIs, they also exhibit various side effects; increased risk of impotence, erectile dysfunction, decreased libido, and ejaculation disorder^32^,^33^.

Therefore, many patients have interests in alternative medicine. In Europe, Asia, and Africa, saw palmetto^34^ and *pyguem aficanum*^35^ are commonly used for treatment of LUTS. Saw palmetto extract is the most famous used herb for phytotherapy in urology. It has an action against 5AR-1 and 2, shows anti-inflammatory effects by inhibition of arachidonic acid metabolites and also has an anti-edematous effect^36^. Traditional Korean Medicine doctors have used a lot of drugs from the past; some studies reported successful effects for BPH by herbal medicines such as Yukmijihwang-tang^37^, *Rubus coreanus*^38^, *Scutellaria baicalensis*^39^, *Curcuma longa*^40^, and *Phellodendron amurense*^41^.

It has been reported that Cinnamomi cortex extracts have a hepatoprotective effect against CCl_4_-induced liver injury^42^ and attenuate atopic dermatitis-like skin lesion induced by mite antigen^43^. CC extracts also have anti-inflammatory effects^44^, as well as antioxidant^45^, gastroprotective^46^, and antimicrobial activities^47^. Although CC has been used to enhance renal function, there is no research for the effects of CC on BPH. In this study, CC effectively reduced prostate size and suppressed the levels of PSA, 5AR-2, ERα, AR, and SRC1 which are closely associated to BPH.

PSA levels can be increased by BPH as well as prostatic inflammation or prostatic cancer. In BPH, immune cells release cytokines and stimulate other cells to release growth factors. Therefore, PSA is a relevant factor which can be used to diagnose BPH. Despite some studies report PSA is not detected in animals besides humans^48^,^49^, several others reported that anti-human PSA antibodies were able to recognize a similar PSA-like protein also in rat prostates^50^,^51^. As shown in Fig. 4, CC effectively decreased the PSA levels in enlarged prostate of the BPH rats.

**Figure 7:**
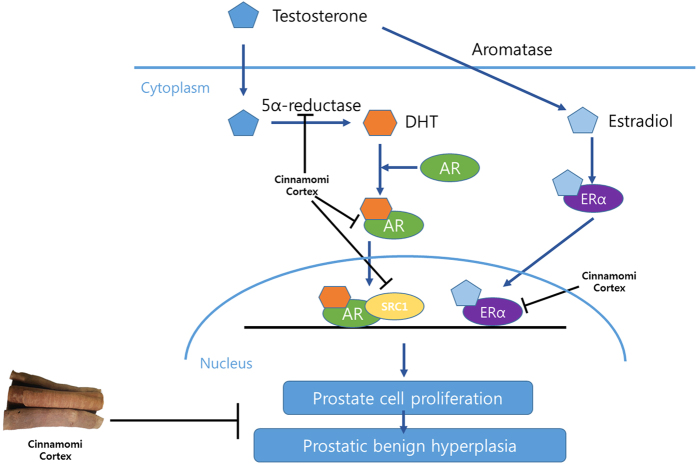
A suggested scheme of the effect of CC on related receptors and coactivators in the pathogenesis of BPH. Testosterone is converted into dihydrotestosterone (DHT) by the action of 5α-reductase (5AR). While DHT binds to the androgen receptor (AR), steroid receptor coactivator 1 (SRC1), a classical type I AR co-regulator, is recruited to the DHT-AR bind. On the other hand, aromatase converts testosterone into estradiol, which also forms a bind with the estrogen receptor α (ERα). The DHT-AR-SRC1 complex and estradiol-ERα bind enter the nucleus and acts as transcription factors, resulting in proliferation of the prostate cells. During the described pathogenesis of benign prostatic hyperplasia (BPH), Cinnamomi Cortex (CC) inhibits 5AR, AR, SRC1 and ERα, suggesting its possibility for BPH treatment.

5AR is an enzyme called 3-oxo-5α-steroid 4-dehydrogenases and involved in steroid metabolism. 5AR is involved in three metabolic pathways; bile acid biosynthesis, androgen and estrogen metabolism, and prostate cancer. 5AR is not only made in the testis and prostate but also in the skin and nervous system. Three types of 5ARs are reported; in fetal period, 5AR-1 is expressed in skin especially the scalp and non-genital skins, while 5AR-2 is expressed in fetal prostates. After birth, the 5AR-1 is expressed in the liver, skin, scalp, and prostate. On the other hand, 5AR-2 is expressed in the prostate, seminal vesicles, and epididymis liver. The main function of 5AR is converting testosterone into DHT. Although the pharmacological mechanism of 5AR inhibition is complex, the inhibition of 5α-reductase results in decreased conversion of testosterone to DHT by reducing the Δ4,5 double-bond^52^. During the binding process of DHT and AR, certain coactivators such as SRC1 are recruited to the DHT-AR bind. Co-regulators are proteins that interact directly with AR to enhance or reduce its activity. SRC1 is a classical type I AR co-regulator^53^ which enhances BPH symptoms. Whereas the role of androgens in BPH has been extensively studied for decades, the effect of estrogens has only recently gained attention. Various estrogens are involved in the prostate growth. Since the early speculations about the potential role of ERs in prostate biology^54^, the effects of estrogens in BPH are recently reviewed, and gaining renewed interest. Different expression patterns and localizations of ERα and ERβ in the prostate make them able to activate or repress the growth control pathways^12^. While ERα leads to proliferation of the prostate, inflammation, and dysplasia, while ERβ suppresses the proliferation of the prostate, inflammation, and dysplasia. As a result, an increased expression of ERα and a decreased expression of ERβ are observed in BPH patients^10^. According to a recent study by Bonkhoff and Berges, increased expression of ERα with the concomitant decrease of ERβ has been shown to correlate with BPH and other prostate diseases^55^. In this study, CC had significant suppressive effect on 5AR, and was also able to down-regulate the level of AR and its co-activator, SRC1. Because 5AR is an important trigger creating prostatic hyperplasia and AR is the main receptor related to 5AR and DHT, these results suggest a potential pharmaceutical therapy of CC on BPH treatment. The authors also investigated the effect of ERα on BPH and demonstrated that CC down-regulates the ERα expression. The suggested scheme of the effects of CC on BPH is shown in Fig. 7.

Further *in vitro* studies using prostate epithelial cells were carried on in order to assess the effect of CC in cellular levels. While the MTS cell proliferation assay showed CC inhibited the proliferation of RWPE-1 cells, additional western blot assays revealed CC treatment successfully decreased the expressions of PSA, 5AR and AR, even when cell proliferation was not inhibited, may be due to the low dose. The *in vitro* results suggest the ability of CC to reduce prostatic proliferation by regulating 5AR, consistent with former *in vivo* experiments.

In this present study, the authors have shown that CC has beneficial effects on the TP-induced BPH rat model. The weight of prostate tissue and epithelial thickness were recovered by up to the level of normal prostate group by CC treatment. Histological changes made by TP inducement were restored similar to the normal group in the CC-treated group. CC-treated group showed significant reductions in PSA and 5AR-2, which implies the restoration to the normal prostate tissue. In addition, the main BPH-related receptors, ERα, AR, and the AR-coactivating protein SRC1, which were all up-regulated in the TP group, were decreased by CC treatment. Furthermore, *in vitro* assays showed CC inhibited cell proliferation by regulating 5AR and AR. These results indicate that CC has positive effects on prostate hyperplasia and suggest a potential of CC for a new pharmacotherapy treatment for BPH.

## Methods

### Sample collection

The bark of *Cinnamomum verum* (Cinnamomi cortex), known as “true cinnamon tree” in English and also called as the name “yukgye” in Korea, was purchased from E-Pul-Lip Pharmaceutical, Co. (Seoul, Republic of Korea). A voucher specimen of the plant has been deposited in our laboratory in Kyung Hee University, Korea. CC was obtained by boiling the herbs in distilled water (1: 7.5, w/v) at 100 °C for 2h. The solution was freeze-dried (yield, 4%). The boiled extract was filtered through a 0.22 μm syringe filter, evaporated, and then stored at −20 °C until usage.

### Chemical reagents

TP was provided from Wako pure chemical industries, and Fi (≥97% pure) was purchased from Sigma-Aldrich Inc. Antibodies for AR, ERα, and SRC1 were purchased from Pierce biotechnology, antibodies for 5AR-2 was products from Abcam Inc.

### GC-MS analysis

The GC-MS was performed on an Agilent 6890N GC system interfaced with Leco Pegasus III GC-TOF Mass Spectrometer. The electron energy was –70 eV, and the ion source temperature was 220 °C. Each sample (1 μl) was injected in split mode (50:1) at 280 °C and separated through a capillary column of INNOWAX (30 × 0.25 × 0.25) (Agilent J&W column). The initial oven temperature was 30 °C, which was increased to 280 °C at 10 °C/min. Carrier gas flow was 0.8 ml/min.

### Animals

12-week-old male Sprague-Dawley (SD) rats (*n* = 56) with initial body weights of 200–220 g were purchased from the Dae-Han Experimental Animal Center. The rats were housed in a pathogen-free room maintained at 23 ± 2 °C and a relative humidity of 70% with an alternating 12 h light/dark cycle. Water and standard laboratory diet (CJ Feed Co., Ltd.) were provided ad libitum. All animal experiments were performed in accordance with the ethical guidelines of Kyung Hee University and approved by the Institutional Review Board of Kyung Hee University (confirmation number: KHUASP (SE)-P-034).

### Experimental procedures

The prostatic hyperplasia model was designed based on Kato’s study^25^. Briefly, BPH was induced by a pre-4-week treatment of daily subcutaneous injections of TP (5 mg/kg) at the inguinal region (*n* = 42). In order to set a control group, 14 rats received ethanol with corn oil instead of TP. After the pre-treatment of 4 weeks, the BPH induced rats were randomly divided into three groups, and the rats those did not receive TP treatment became the normal control group. Thus, the rats were divided into four groups: (a) a normal control group that received ethanol with corn oil; (b) a BPH group that received TP with corn oil; (c) a positive control group that received Fi (1 mg/kg), a 5ARI which is frequently used as treatment for BPH, with TP (5 mg/kg); and (d) a group that received CC (5 mg/kg) with TP (5 mg/kg). CC and Fi were administered to animals once daily for 4 weeks, following the pre-4-week BPH inducement. The animals were sacrificed at the end of week 4. After the final treatment, animals were fasted overnight and euthanized using CO_2_. Blood samples were obtained from the caudal vena cava. The blood containing tubes were remained at RT for 2 h and sera were separated by centrifuging at 3,000× *g* for 20 min in 4 °C. The serum was stored at −80 °C until further assays. The intact prostate tissue was carefully dissociated and removed, washed with PBS, and then weighed. Relative prostate weight was calculated as the ratio of prostate weight (mg) to body weight (100 g). Percent recovery was calculated as follows;


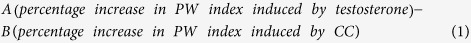


Percentage of inhibition of increase in PW index was calculated as follows;


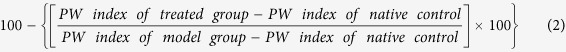


The prostate tissue was divided in half; one half was fixed in 10% formailin and embedded in paraffin for histomorphological assays, the other was stored at −80 °C for further assays.

### Hematoxylin and eosin (H&E) staining and immunohistochemistry (IHC)

The formalin-fixed, paraffin-embedded prostate specimens were cut into 4-μm-thick tissue sections. The sections were deparaffinized in xylene and rehydrated in serial alcohol. After treatment of 150 μl 0.1% trypsin working solution (consisted of trypsin 0.4 ml, calcium 0.01 g, chloride 0.01 g in D.W. 7 ml) for 15 min, the sections were blocked using fetal bovine serum (FBS). For H&E staining, the sections were stained in hematoxylin for 5 min, and then washed with water for 5 min. Then the sections were stained in eosin for 30 s, dehydrated, and mounted by routine methods. For immunostaining, sections were incubated in 4 °C overnight with a 1:50 dilution of the primary antibody AR and ERα; then incubated at room temperature for 30 min with a 1:500 dilution of the horseradish peroxidase (HRP)-conjugated affinipure Goat anti-rabbit IgG (Jackson Immunoresearch lab.) or HRP-conjugated affinipure Goat anti-mouse IgG (Jackson Immunoresearch lab.). Following the addition of the detection system, the reaction was visualized using diaminobenzidine (DAB) in the presence of hydrogen peroxide. The slides were examined using the Olympus IX71 Research Inverted Phase microscope (Olympus Co.), and the density was measured using ImageJ 1.47 v software (National Institute of Health).

### Western blot assay

Prepared prostate tissues were cut into pieces and homogenized by using the Bullet Blender homogenization kit (Next Advance Inc.). Homogenized tissues or harvested cells were lysed with ice-cold RIPA buffer and then centrifuged at 13,000 rpm for 20 min at 4 °C to remove the insoluble materials. Next, the total concentration of extracted proteins was determined using the method of Bradford^56^ as in the authors’ previous studies^57^,^58^,^59^. Briefly, the proteins in the supernatants were separated by 8% sodium dodecyl sulfate-polyacrylamide gel electrophoresis (SDS-PAGE) and transferred onto polyvinylidenedifluoride (PVDF) membranes. After blocking with 10 mM Tris, 150 mM NaCl, and 0.05% Tween-20 (TBST) (pH 7.6) containing 5% skim milk for 1 hour at room temperature, the membranes were washed with TBST and then incubated with the appropriate primary antibodies (AR, SRC1 or 5AR) at 4 °C overnight. The blots were subsequently incubated with HRP-conjugated affinipure Goat anti-rabbit IgG (Jackson Immunoresearch lab.) or HRP-conjugated affinipure Goat anti-mouse IgG (Jackson Immunoresearch lab.). PVDF membrane was purchased from Millipore, and the protein assay reagent was obtained from Bio-Rad. The chemiluminescent intensities of protein signals were quantified using ImageJ 1.47 v software (National Institute of Health).

### Cell culture

The normal human prostatic epithelial cell line RWPE-1 was obtained from the American Type Culture Collection. RWPE-1 cells were cultured in Roswell Park Memorial Institute medium (RPMI) (Gibco) supplemented with 100 mg/ml penicillin/streptomycin (Hyclone) and 10% fetal bovine serum (FBS) (Sigma).

### MTS assay

RWPE-1 cells were seeded (3 × 10^4^ cell/well) and incubated in RPMI plus 10% FBS for 24 h. Then the cells were incubated in fresh media containing CC for an additional 24 h. Cell viability was monitored using the cell proliferation MTS kit by the Promega Corporation as recommended by the manufacturer. Prior to measuring the viability, the media were removed and replaced with 200 μl of fresh RPMI plus 10% FBS medium and 10 μl of 3-(4,5-dimethylthiazol-2-yl)-5-(3-carboxymethoxyphenyl)-2-(4-sulfophenyl)-2*H*-tetrazolium (MTS) solution. The cells were then incubated in the incubator for 4 h. The absorbance was measured at 490 nm in a VERSAmax microplate reader (Molecular Devices) to determine the formazan concentration, which is proportional to the number of live cells.

### Statistical analysis

All results were expressed as the mean ± S.D. Differences in mean values were analyzed by one-way ANOVA or one-tailed Student’s *t* test using IBM SPSS Statistics 22 software (International Business Machines Corp.). Values with *P* < 0.05 were considered to indicate statistical significance.

## Additional Information

**How to cite this article**: Choi, H.-M. *et al*. Cinnamomi Cortex (*Cinnamomum verum*) Suppresses Testosterone-induced Benign Prostatic Hyperplasia by Regulating 5α-reductase. *Sci. Rep.* **6**, 31906; doi: 10.1038/srep31906 (2016).

## Supplementary Material

Supplementary Information

## Figures and Tables

**Table 1:** Effect of CC on prostatic parameters.

Group	Body weight (g)	PW (mg)	PW Index (mg per body weight 100 g)	Increase in PW Index (%)	Inhibition of Increase in PW index (%)	Recovery rate (%)
**B**	473.1 ± 15.6	635 ± 143	111 ± 18.3	—	—	—
**C**	443 ± 41.2	1457 ± 196^#^	339.4 ± 64.8^#^	100	—	—
**CC**	438 ± 36.9	798 ± 217^**^	173.7 ± 56.1^*^	51.2	72.8	48.8
**Fi**	452.5 ± 34.4	823 ± 332^**^	162.3 ± 20.8^**^	47.8	77.6	52.2

Values are mean ± S.D. of data from three separate experiments. ^#^*P* < 0.05 when compared to B; *P < 0.05 when compared to C. PW, prostate weight; B, normal control group; C, TP-induced BPH group; CC, CC treated BPH group; Fi, Fi treated BPH group.
