# Supplementary material for: Cinnamomi Cortex (Cinnamomum verum) Suppresses Testosterone-induced Benign Prostatic Hyperplasia by Regulating 5α-reductase
Source: Sci Rep. 2016 Aug 23;6:31906. doi: 10.1038/srep31906 (PMC4994048; doi:10.1038/srep31906)
Supplement: Supplementary Information [file srep31906-s1.pdf]

Cinnamomi Cortex (*Cinnamomum verum*) Suppresses Testosterone-induced Benign Prostatic Hyperplasia by Regulating 5α-reductase

Hyun-Myung Choi[1,+], Yunu Jung[2,+], Jinbong Park[2,+], Hye-Lin Kim[1], Dong-Hyun Youn[2], JongWook Kang[2], Mi-Young Jeong[1], Jong-Hyun Lee[3], Woong Mo Yang[1], Seok-Geun Lee[1], Kwang Seok Ahn[1], and Jae-Young Um[1,2,*]

Supplementary Information

Supplementary Figure S1

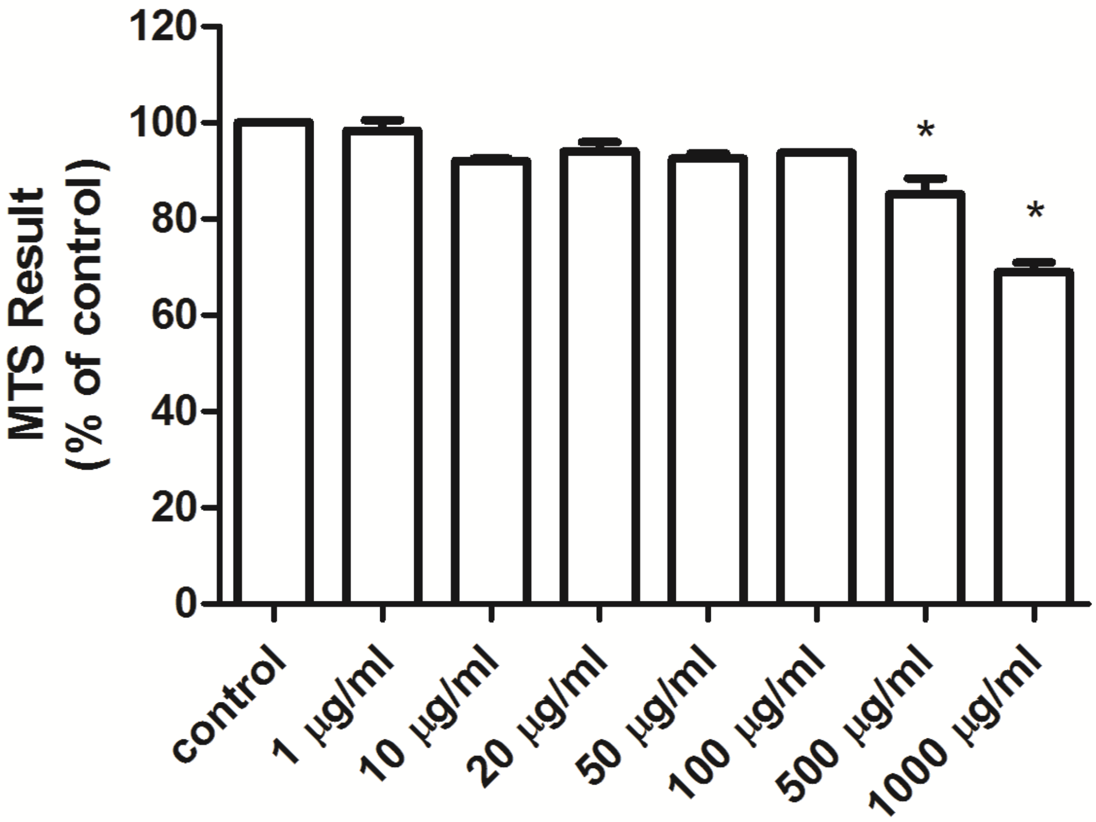

Supplementary Figure S1. Cell proliferation test of CC in RWPE-1 cells. The normal human prostatic epithelial cell line, RWPE-1, were treated with various concentrations of CC (1 – 1000 μg/ml) for 24 h, and then the cell proliferation was determined by an MTS assay. Data are expressed as mean ± S.E.M. of three or more experiments. $^{*}p < 0.05$ vs. non-treated control RWPE-1 cells.

Supplementary Figure S2

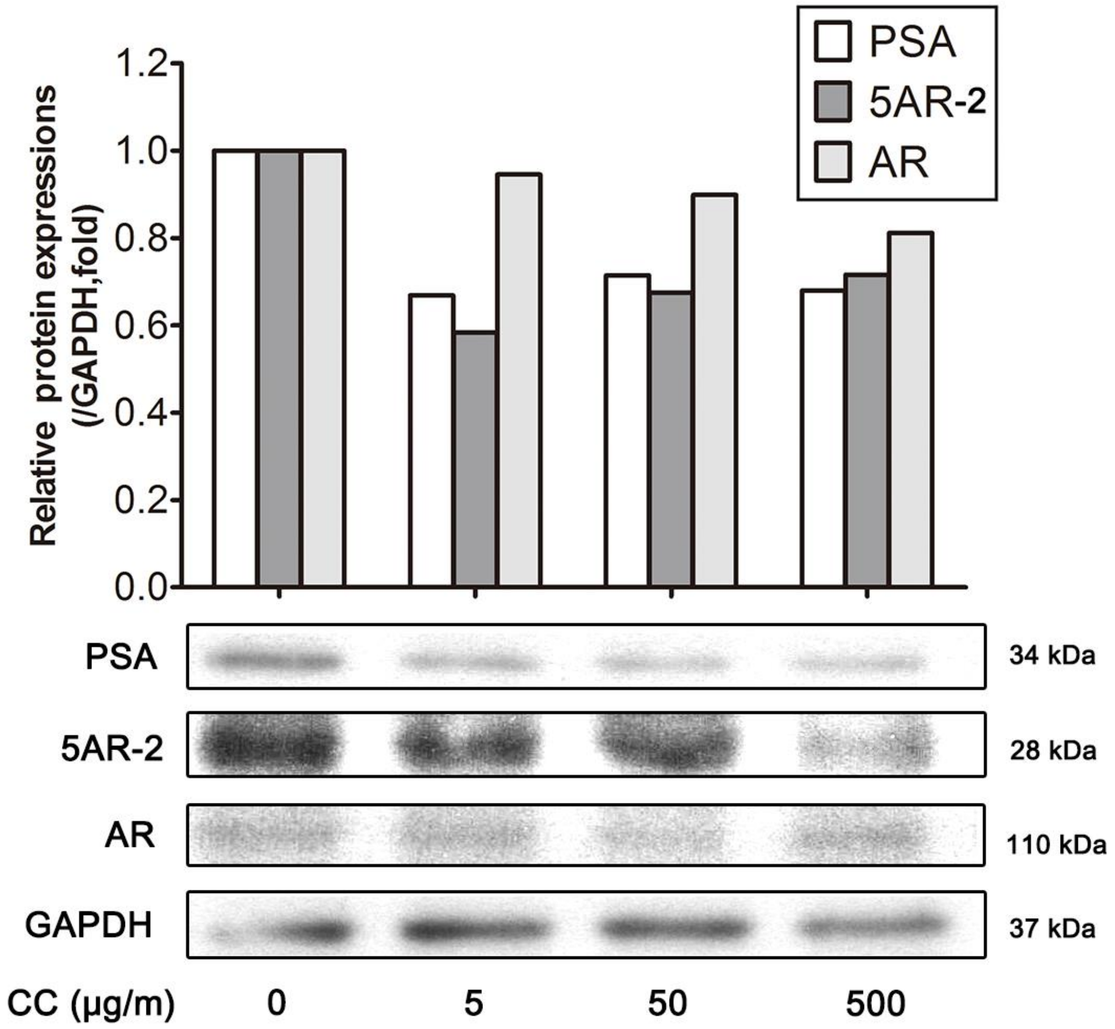

Supplementary Figure S2. Effect of CC on BPH-related protein expressions in RWPE-1 cells. The normal human prostatic epithelial cell line, RWPE-1, were treated with various concentrations of CC (5, 50 and 500 μg/ml) for 24 h, and then the expressions of PSA, 5AR-2 and AR were measured using a western blot assay. GAPDH was used as an endogenous control.
